# Effect of Air Exposure-Induced Hypoxia on Neurotransmitters and Neurotransmission Enzymes in Ganglia of the Scallop *Azumapecten farreri*

**DOI:** 10.3390/ijms23042027

**Published:** 2022-02-11

**Authors:** Elena Kotsyuba, Vyacheslav Dyachuk

**Affiliations:** A.V. Zhirmunsky National Scientific Center of Marine Biology, Far Eastern Branch, Russian Academy of Sciences, 69004 Vladivostok, Russia; epkotsuba@mail.ru

**Keywords:** stress, neurotransmitters, biogenic amines, bivalves, oxygen

## Abstract

The nervous system expresses neuromolecules that play a crucial role in regulating physiological processes. Neuromolecule synthesis can be regulated by oxygen-dependent enzymes. Bivalves are a convenient model for studying air exposure-induced hypoxia. Here, we studied the effects of hypoxia on the expression and dynamics of neurotransmitters, and on neurotransmitter enzyme distribution, in the central nervous system (CNS) of the scallop *Azumapecten farreri*. We analyzed the expression of the neurotransmitters FMRFamide and serotonin (5-HT) and the choline acetyltransferase (CHAT) and universal NO-synthase (uNOS) enzymes during air exposure-induced hypoxia. We found that, in early-stage hypoxia, total serotonin content decreased in some CNS regions but increased in others. CHAT-lir cell numbers increased in all ganglia after hypoxia; CHAT probably appears de novo in accessory ganglia. Short-term hypoxia caused increased uNOS-lir cell numbers, while long-term exposure led to a reduction in their number. Thus, hypoxia weakly influences the number of FMRFamide-lir neurons in the visceral ganglion and does not affect peptide expression in the pedal ganglion. Ultimately, we found that the localization and level of synthesis of neuromolecules, and the numbers of cells expressing these molecules, vary in the scallop CNS during hypoxia exposure. This indicates their possible involvement in hypoxia resistance mechanisms.

## 1. Introduction

Many invertebrates, such as marine bivalves, have adapted to reduced concentrations (hypoxia) or a total lack of oxygen (anoxia) during their evolution [1,2,3]. However, little is known about the mechanisms of their resistance to hypoxia or their survival strategy. An effective mechanism by which mollusks adapt to stress, including hypoxia caused by any significant environmental changes, is the neuroendocrine stress response [4,5,6]. Such a response involves variations in the physiology of the organism and adaptive changes in behavior due to the processes induced by stress factors in the nerve ganglia. Various hormones, referred to as neurotransmitters, as well as their receptors and key enzymes for their synthesis, that have been identified in invertebrates to date, have a molecular basis similar to those in vertebrates and are involved in homeostatic regulation [7,8,9].

Hypoxia-sensitive species are distinguished from tolerant ones by their regulation of physiological and metabolic processes and their behavioral adaption [1,2] to hypoxia, including prompt organization of various adaptive locomotor responses. These species include the scallop *Azumapecten farreri*, which is a steno-oxyphilic organism sensitive to low-oxygen conditions and lives in relatively stable habitats of the subtidal zone. It is usually found attached to rocks and other substrates via its byssus threads but can also move to new habitats under adverse conditions. A characteristic protective behavioral response of scallops to stressful conditions (such as abnormal temperature, predation pressure, or low oxygen concentrations in seawater) is escape facilitated by contraction of the adductor muscle [10,11,12], which is crucial for survival. This type of behavior is known to be associated with discrete signaling systems. Therefore, it can be hypothesized that a forced restriction of movements would induce significant changes in the metabolism of neurotransmitters and neurohormones.

One of the most important neurotransmitters involved in the regulation of locomotor responses in mollusks is serotonin (5-hydroxytryptamine, 5-HT) [13,14]. 5-HT and its receptors have been identified in the CNS of vertebrates and all groups of invertebrates, including nematodes [15], annelids [16], arthropods (insects and crustaceans) [17,18], and mollusks [19,20,21,22,23]. 5-HT performs an important role in the organization of various behavior patterns in invertebrates [24]. A variation in the level of 5-HT or a disturbance in its synthesis or metabolism can result in the alteration of the behavioral and locomotor responses exhibited by these animals [18].

The role of the cholinergic neurotransmitter system in the regulation of locomotor activity in invertebrates and vertebrates has been highly researched [25]. There are many indications that the acetylcholine (ACh) neurotransmission system found in vertebrates is also present in mollusks. Biochemical and histochemical studies have demonstrated the action of the enzyme synthesizing acetylcholine (choline acetyltransferase, CHAT) in multiple different taxonomic groups of mollusks [26,27,28,29,30,31]. The involvement of ACh in the contraction of the anterior byssus retractor, the pharynx elevator, the radula protractor, the gill, and the buccal and swim muscles has been shown experimentally [32,33,34]. Cholinergic motor neurons involved in locomotor responses in mollusks have also been identified [35,36].

In addition to the above-mentioned neurotransmitters, the involvement of nitric oxide (NO) in the regulation of the activity of motor neurons in mollusks has been shown [37]. Nitric oxide is synthesized via the enzyme NO-synthase (NOS). The NOSs that have been identified in gastropods, cephalopods, and bivalves, including scallops [38,39,40,41,42,43], all belong to a single isoform. NO-positive neurons and nerve fibers have been detected in the nerve ganglia, in the peripheral system (in various parts of the intestinal tract), in female and male gonads, and in the hemocytes of marine, freshwater, and terrestrial mollusks [44,45,46,47]. Several studies have demonstrated the involvement of NO-signaling in stress responses in cases of exposure to heat stress [48], increased temperature [49], and environmental pollution [47,50].

FMRFamide-like peptides have been described in all the major phyla of animals [51,52], including in the central and peripheral nervous systems of various taxonomic groups of mollusks [53,54,55]. It has been reported in recent years that FMRFamide-like peptides in mollusks are involved in the action of the cardiovascular system, modulation of muscle contraction, control of motor activity, and regulation of water homeostasis [53]. Neuropeptides, including FMRFamide-like peptides, are regulators of stress responses; however, variations in their expression during stress responses in invertebrates have not been sufficiently studied to date. The first evidence that neuropeptides and FMRFamide are also involved in adaptation to hypoxic stress in crustaceans has been presented relatively recently [56].

Hypoxia and anoxia are known to cause disturbances in behavioral responses in mollusks, to increase their risk of mortality, and to reduce their chance of survival [57]. Unfortunately, little is known about the role of the neurotransmitters and neuromodulators involved in the stress response in mollusks when their escape behaviors are disrupted. Under experimental conditions, a convenient model for hypoxic stress with disturbed escape behaviors in scallops is water deprivation. These conditions are usually created during the transportation of the animals for the purpose of acclimatization.

In the present work, we study the effects of air exposure-induced hypoxia on the activity of neurotransmitter synthesis enzymes, including CHAT and universal nitric oxide-synthase (uNOS), and the expression of 5-HT and FMRFamide in the CNS of the scallop species *A. farreri*, in the case of disturbances to their escape behaviors. It was found that, under these conditions, the synthesis levels of neurotransmitter enzymes varied, suggesting that these enzymes are involved in the hypoxia stress response in *A. farreri*.

## 2. Results

### 2.1. General Neuroanatomy of the Scallop Azumapecten farreri

**Figure 1:**
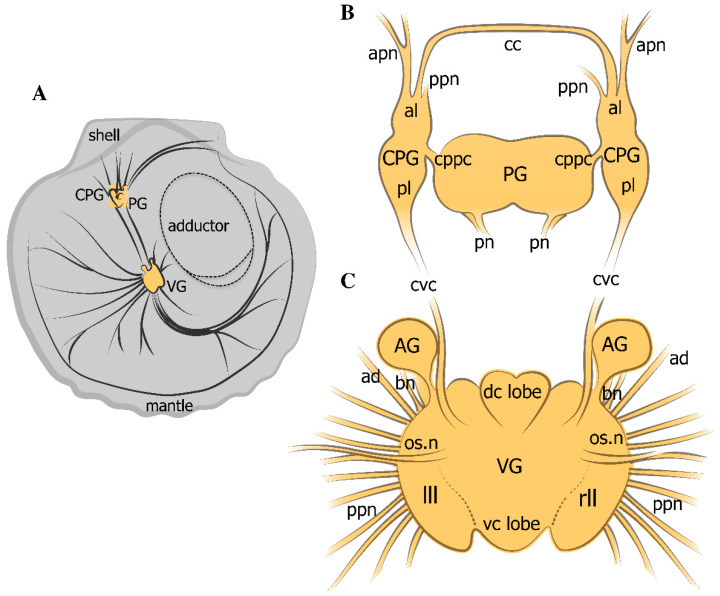
Diagrams of the nervous system in the scallop *Azumapecten farreri*. General view of the mollusk nervous system (**A**). Cerebropleural paired ganglia (CPG) are formed by fusion of the cerebral (anterior lobe) and pleural (posterior lobe) ganglia (**B**). Cerebral commissures (cc) extend from the anterior lobe of the CPG, cerebral–pleural–pedal connectives (cppc) connect to the pedal ganglia (PG), and connectives from the pleural lobe of CPG connect to the fused visceral ganglia (VG) via long cerebral–pleural visceral paired connectives (cpvc). The visceral ganglia consist of four lobes: two lateral (left and right) lobes (lll and rll), dorso-central (dc), and ventro-central (vc) lobes, accessory ganglia (AG), and nerves extending to the periphery, including posterior pallial (ppn), branchial (b.n.), and osphradial nerves (os.n.) (**C**). The sizes of the ganglia are as follows: VG—length 3.5 mm, width 3.0 mm, thickness 1.5 mm; PG—length 1.1 mm, width 0.8 mm, thickness 0.6 mm; paired CPG, each ganglion—length 1 mm, width 0.9 mm, thickness 0.6 mm; cerebral commissure, diameter 0.6 mm; cerebropedal connective—length approximately 1 mm and diameter 0.6 mm; the pedal commissure is shortened to 0.6 mm.

When describing the anatomical organization of the nervous system in *A. farreri*, we have used the terminology accepted by other researchers who have studied this scallop species [58,59,60]. The CNS in the scallop *A. farreri* is one of the most complex nervous systems among that of bivalve mollusks (Figure 1A). It consists of two pairs of ganglia, the cerebropleural ganglia (CPG), fused pedal ganglia (PG) (Figure 1B), and one unpaired visceral ganglion (VG), as well as multiple other nerves (Figure 1C). The CPG, consisting of a cerebral (anterior) lobe and a pleural (posterior) lobe, is located on either side of the pedal ganglion, which lies at the base of the foot (Figure 1). The VG is located on the ventral surface of the striated adductor muscle and is linked to the CPG via the cerebropleuro-visceral connectives. The VG has a complex structure and comprises two dorso-central (anterior), one ventro-central (posterior), and two lateral lobes (Figure 1C). Furthermore, the VG includes two accessory ganglia (AG) located between the cerebro-visceral connective and the branchial nerves (Figure 1C). The AG are connected to the VG, the nerves of which run parallel to the branchial nerve (Figure 1C).

### 2.2. HIF1alpha Protein Expression

**Figure 2:**
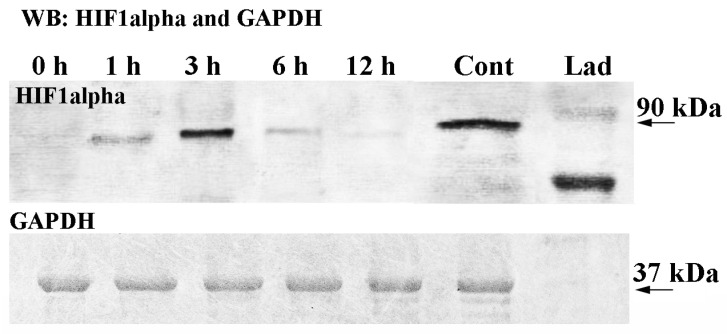
Western blot analysis of HIF1alpha protein levels in scallop *Azumapecten farreri* in total ganglia extracts during air exposure. The glyceraldehyde-3-phosphate dehydrogenase (GAPDH) antibody was used as a loading control.

After SDS-PAGE, the transfer of proteins to the membrane, and the performance of Western blotting, the dynamics of HIF1alpha protein expression became apparent (Figure 2). The HIF1alpha commercial primary antibody recognized mouse HIF1alpha (from the brain, positive control) as well as the HIF1alpha-like protein from a total extract of scallop ganglia (Figure 2). The data showed that HIF1alpha was not detected in the total extract of ganglia from the control samples (0 h exposure or normoxia) (Figure 2). However, after 1 h of air exposure-induced hypoxia (further hypoxia), an HIF1alpha band (*M*_W_ 90 kDa) was clearly visible (Figure 2). The strongest protein signal was detected after 3 h of hypoxia, and then the signal weakened after 6 and 12 h (Figure 2). Thus, the data on the expression of HIF1alpha during hypoxia showed that this timeframe of hypoxia could be used for the analysis of the distribution of neurotransmitters and their enzymes under these conditions in further experiments.

### 2.3. Impact of Hypoxia on Neurotransmitter Distribution in Scallop CNS

#### 2.3.1. 5-HT-1ir Distribution after Hypoxia Exposure

**Figure 3:**
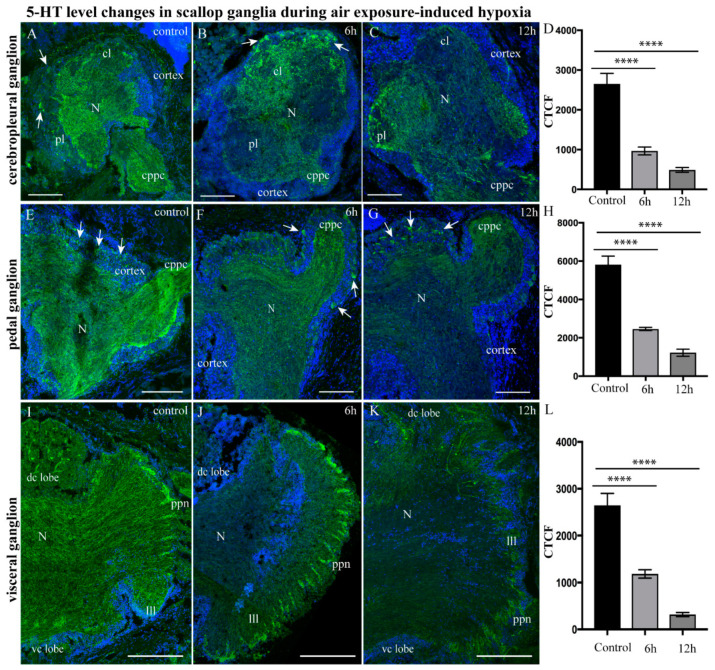
Elevation of serotonin 5-HT-lir level in scallop ganglia during air exposure-induced hypoxia. The distribution of 5-HT immunoreactivity in the CPG under control conditions (**A**) and after 6 h (**B**) and 12 h (**C**) of hypoxia exposure. (**D**) Quantitative analysis of the immunopositive reaction in the CPG. The distribution of 5-HT immunoreactivity in the PG under control conditions (**E**) and after 6 h (**F**) and 12 h (**G**) of hypoxia exposure. The arrows on the figures indicate immunopositive cells. (**H**) Quantitative analysis of the immunopositive reaction in the PG. The distribution of 5-HT immunoreactivity in the VG under control conditions (**I**) and after 6 h (**J**) and 12 h (**K**) of hypoxia exposure. (**L**) Quantitative analysis of immunopositive reaction in the VG. The letter designations are as follows: VG, visceral ganglia; N, neuropil; vc lobe, ventro-central lobe; dc lobe, dorso-central lobe; lll, left lateral lobe; rll, right lateral lobe; ppn, posterior pallial nerves; AG, accessory ganglia; cpvc, cerebral–pleural visceral paired connectives; bn, branchial nerves. Data analysis was performed with GraphPad Prism 7; the data are expressed as mean ± SEM, and statistical significance was calculated using a one-way ANOVA test. Multiple comparisons were performed using Dunnett’s many-to-one test (normoxia group was set as control). Regarding corrected fluorescence intensity (CTFC) reduction in CPG after hypoxia, F = 46.78, *p* < 0.0001, R square = 0.7958, F (DFn, DFd): F (2, 24) = 46.78. In PG after hypoxia, F = 73.33, *p* < 0.0001, R square = 0.8544, F (DFn, DFd): F (2, 25) = 73.33. For VG after hypoxia, F = 55.63, *p* < 0.0001, R square = 0.8226, F (DFn, DFd): F (2, 24) = 55.63. Degree of significance is represented as follows: **** *p* < 0.0001. Bars: (**A**–**G**), 100 μm; (**I**–**K**), 200 μm.

In *A. farreri*, 5-HT-lir was present in all ganglia (Figure 3), but the serotonin distribution and the intensity of staining differed between the ganglia of the control animals and those subjected to air exposure-induced hypoxia (further hypoxia) (Figure 3A–L). In the cerebral and pleural lobes of the CPG of the control group, single 5-HT-lir neurons were labeled in the cell body layer (CBL) of the ganglion, and numerous thin varicose fibers were labeled in neuropils (Figure 3A). After 6 h of hypoxia exposure, a significant decrease in the level of 5-HT-lir was recorded in the pleural lobe of the CPG, whereas the immunoreactivity in the cerebral lobe was slightly higher than that of the control group (Figure 3B). After 12 h of hypoxia exposure, the level of 5-HT-lir decreased in all regions of the CPG (Figure 3C). With regards to 5-HT, 1 and 3 h hypoxia had no effect on the distribution of serotonin in the ganglia (either on the number of cells or on the amount of serotonin in neurites) compared to the control.

A quantitative analysis of corrected fluorescence intensity (CTCF) showed a decrease in the fluorescence signal after 6 h (964.89 ± 98.47) and 12 h of hypoxia exposure (489.70 ± 59.43) in the CPG compared to that of the control ganglia (2651.94 ± 386.31) (Figure 3D). In the control PG, 5-HT-lir neurons were detected in the CBL, and a dense network of 5-HT-lir fibers was identified in the lateral region of each ganglion and in the cerebropleuropedal connectives (Figure 3E). In the PG, a decrease in the level of 5-HT-lir was detected compared to that of the control ganglion (5814.83 ± 446.55) after just 1 h of hypoxia exposure (data not provided). This level continued to decrease after 6 h (2436.46 ± 86.97), reaching a minimum value by 12 h (1223.09 ± 184.48) (Figure 3F–H). In the control VG, 5-HT-lir was detected in the varicose fibers of the neuropil, the cerebrovisceral connectives, and the peripheral nerves (Figure 3I). No 5-HT-lir bodies of neurons were found in the VG (Figure 3I). After 6 h of hypoxia exposure, the level of 5-HT immunoreactivity decreased in the neuropils of the entire ganglion, only remaining in the nerves extending from the ganglia (Figure 3J). After 12 h of hypoxia exposure, the level of 5-HT-lir in all ganglia decreased below the control level (Figure 3K). A quantitative analysis of the fluorescent activity showed a decrease in the serotonin concentration compared to the control value (2644.83 ± 254.60) after 6 h (1185.04 ± 88.69) and 12 h (317.97 ± 43.43) of hypoxia exposure (Figure 3L).

Thus, in the early stages, hypoxia in scallops causes the amount of total serotonin to decrease in all ganglia, while longer hypoxia exposure leads to reductions in serotonin levels in the cortex ganglia, neuropils, and in the nerves extending from the ganglia.

#### 2.3.2. Choline Acetyltransferase-lir Distribution after Hypoxia Exposure

In the normoxic scallops (control), CHAT-lir was detected in the cell bodies of the cortex of the anterior (cerebral) lobe and the neuropil of the CPG (Supplementary Figure S1A–A2), as well as in the neuropil of the PG and in the fibers of cerebropleuropedal connectives running from the PG (Supplementary Figure S1B). After 1 h of hypoxia exposure, the number of CHAT-lir neurons remained almost unchanged in CPG (Supplementary Figure S1). However, after 12 h, the proportion of CHAT-lir neurons in the CPG and the PG grew significantly (CPG 27.09 ± 2.15%; PG 7.30 ± 0.47%). This was much higher than that of the short-term hypoxia exposure group (1 h) (CPG 3.41 ± 0.18%; PG 0.15 ± 0.04%) and control group (CPG 3.09 ± 0.28%; PG 0 ± 0%) (Supplementary Figure S1C–F). However, 3 and 6 h of hypoxia had no effect on the distribution of CHAT in the CPG and PG.

**Figure 4:**
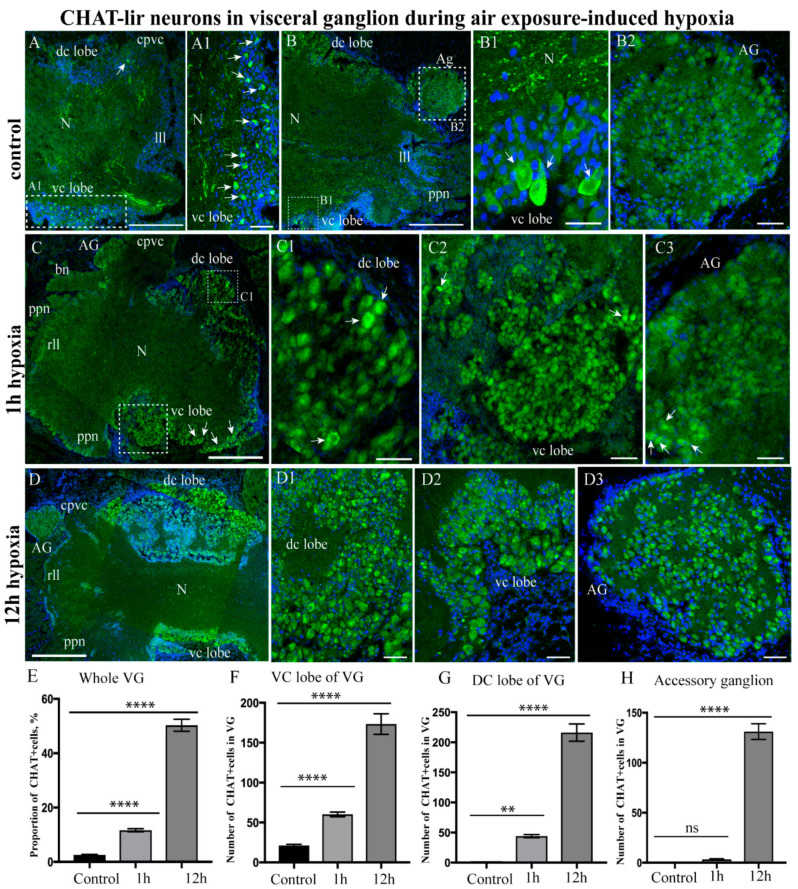
Changes in choline acetyltransferase (CHAT)-lir distribution in the visceral ganglion (VG) during air exposure-induced hypoxia. The distribution of CHAT-lir in the VG under control conditions (**A**–**B2**) and after 1 h (**C**–**C3**) and 12 h of hypoxia exposure (**D**–**D3**). (**E**) Quantification of CHAT-lir cells in the VG (proportion of CHAT+ cells, %) in control ganglia and after hypoxia exposure. (**F**–**H**) The total number of CHAT-lir cells in various VG parts ((**F**), visceral lobe; (**G**), dorsal lobe; (**H**), accessory ganglion) under control conditions and after hypoxia exposure (1 and 12 h). The arrows on the figures indicate immunopositive cells. The letter designations are as follows: VG, visceral ganglion; N, neuropil; vc lobe, ventro-central lobe; dc lobe, dorso-central lobe; lll, left lateral lobe; rll, right lateral lobe; ppn, posterior pallial nerves. Data analysis was performed with GraphPad Prism 7; the data are expressed as mean ± SEM, and statistical significance was calculated using one-way ANOVA tests. Multiple comparisons were performed using Dunnett’s many-to-one test (the normoxia group was set as the control). Regarding the proportion of CHAT+ cells in the whole VG: F = 364.5, *p* < 0.0001, R square = 0.9681, F (DFn, DFd): F (2, 24) = 364.5. With regard to the number of CHAT+ cells in the visceral lobe of the VG: F = 107, *p* < 0.0001, R square = 0.8991, F (DFn, DFd): F (2, 24) = 107; for the dorsal lobe of the VG: F = 180.6, *p* < 0.0001, R square = 0.9377, F (DFn, DFd): F (2, 24) = 180.6; for the AG: F = 275.1, *p* < 0.0001, *p* = 0.8331, R square = 0.9582, F (DFn, DFd): F (2, 24) = 275.1. The degree of significance is represented as follows: **** *p* < 0.0001, ** *p*-value ≤ 0.01; ns—non-significant *p*-value. Bars: (**A**,**B**), 100 μm; (**C**,**D**), 200 μm; (**B1**,**B2**,**C1**–**C3**,**D1**–**D**), 20 μm.

As for control VG, large solitary (20–30 μm) CHAT-lir neurons were detected in the dorso-central lobes and in large aggregations in the ventro-central lobes (Figure 4A Panel A1,B,B1). Moreover, CHAT-lir was detected in the VG fibers of the neuropil and in the nerves extending from the ganglia (Figure 4A), but not in the control AG (Figure 4 Panel B2). Under hypoxic conditions, we observed the relationship of the CHAT-lir level in various parts of the VG with the length of exposure. In the VG, the CHAT-lir level in the dorso- and viscero-central lobes, as well as in neurons localized in the lateral lobes, increased after 1 h of hypoxia exposure (Figure 4 Panel C–C3). Large CHAT-lir neurons with no histochemical activity in the control were labeled in the ventro-central lobes and AG (Figure 4 Panel C,C2). In the VG, the CHAT-lir level was remarkably increased in the neurons of the dorso-central (Figure 4 Panel D,D1) and ventro-central lobes after 12 h of hypoxia exposure (Figure 4 Panel D,D2). Furthermore, the intensity of staining in the neurons increased in the AG (Figure 4 Panel D3).

The quantitative assessment of CHAT-lir neurons in the VG revealed that there was an increase in the proportion of immunopositive neurons after 1 h of hypoxia exposure (11.66 ± 0.56%) and an even greater increase after 12 h (50.30 ± 2.21%), compared to that in the control (2.52 ± 0.22%) (Figure 4E). Of particular interest is the observation that the numbers of CHAT-lir neurons increased simultaneously in the dorso-central (44 ± 2.63 cells) and ventro-central (60 ± 2.85 cells) lobes of the VG after 1 h of hypoxia exposure and also in the dorso-central (216.11 ± 14.43 cells) and ventro-central (173.33 ± 12.84 cells) lobes of the VG after 12 h of hypoxia exposure, compared to those in control conditions (dorso-central lobes, 0.88 ± 0.26 cells; ventro-central lobes, 21.22 ± 1.40 cells) (Figure 4F,G). Moreover, solitary CHAT-lir cells appeared in the AG after only 1 h of hypoxia (Figure 4 Panel C3). The number of CHAT-lir cells increased in the AG as the period of exposure to hypoxia lengthened and reached a maximum at 12 h (131.11 ± 7.78 cells) (Figure 4H). In contrast, CHAT-lir cells were completely absent in the control (Figure 4H).

Thus, hypoxia causes the number of CHAT-lir neurons to increase in all ganglia, while CHAT-lir neurons in the PG and AG probably appear de novo, as normoxic (control) ganglia lack these neurons.

#### 2.3.3. uNOS-lir Distribution after Hypoxia Exposure

**Figure 5:**
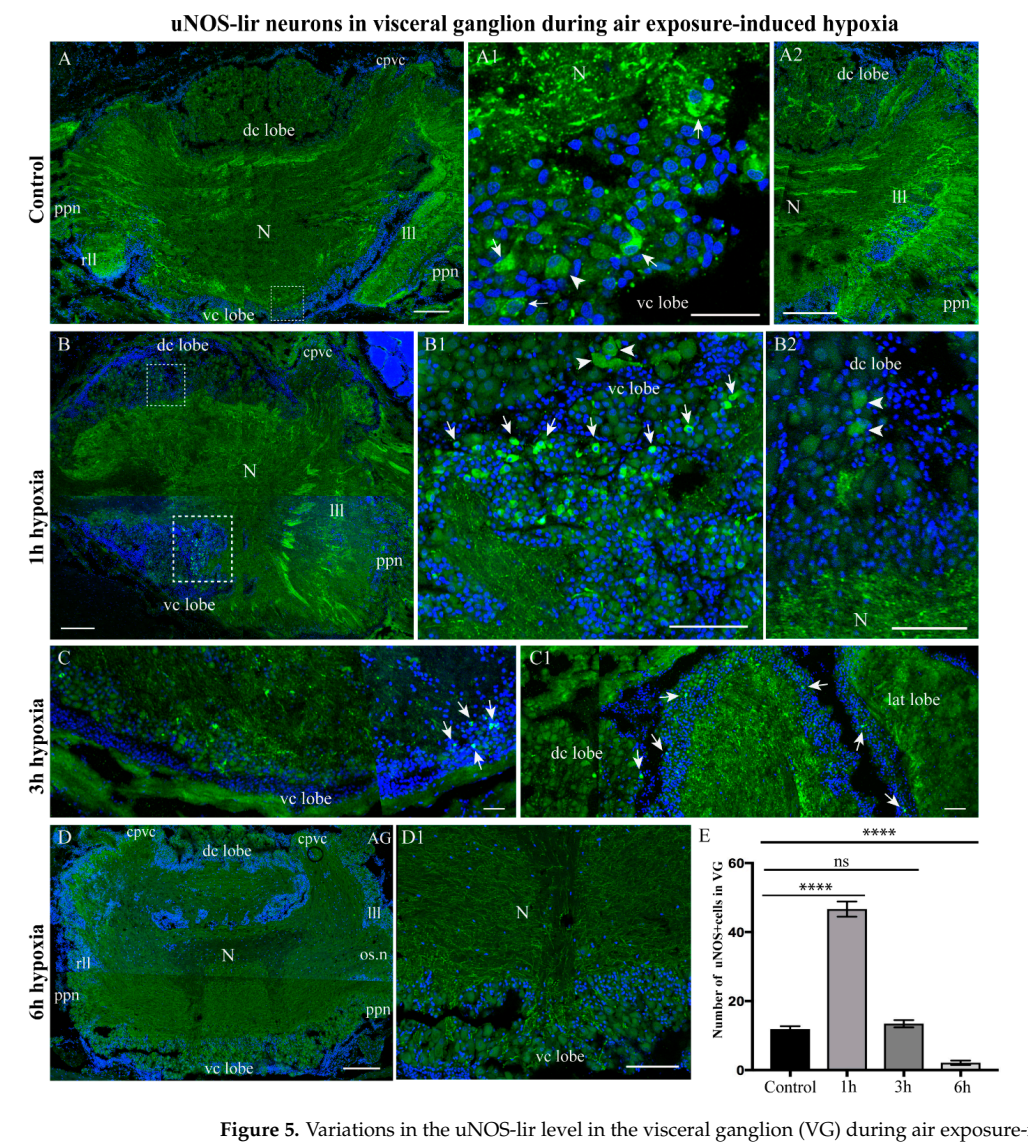
Variations in the uNOS-lir level in the visceral ganglion (VG) during air exposure-induced hypoxia. The distribution of uNOS-lir neurons in the VG under control conditions (**A**–**A2**) and after 1 h (**B**–**B2**), 3 h (**C**,**C1**), and 6 h (**D**,**D1**) of hypoxia exposure. The arrows on the figures indicate immunopositive cells. (**E**) Quantification of uNOS-lir neurons in the VG. The letter designations are as follows: AG, accessory ganglia; CPG, cerebropleural ganglia; PG, pedal ganglia; VG, visceral ganglia; N, neuropil; vc lobe, ventro-central lobe; dc lobe, dorso-central lobe; lll, left lateral lobe; rll, right lateral lobe; cppc, cerebral–pleural–pedal connectives; cpvc, cerebral–pleural visceral connectives; ppn, posterior pallial nerves. Data analysis was performed with GraphPad Prism 7; the data are expressed as mean ± SEM, and statistical significance was calculated using one-way ANOVA tests. Multiple comparisons were performed using Dunnett’s many-to-one test (normoxia group was set as the control). Regarding the proportion of NOS+ cells in the VG: F = 213.8, *p* < 0.0001 (****), *p* = 0.7455, R square = 0.9525, F (DFn, DFd): F (3, 32) = 213.8. The degree of significance is represented as follows: **** *p* < 0.0001; ns—non-significant *p*-value. Bars: (**A**,**B**,**D**) 100 μm; (**A1**), 20 μm; (**B1**,**B2**,**C**,**C1**,**D1**), 50 μm.

In the control *A. farreri* scallops, uNOS-lir neurons were not detected in the CPG (Supplementary Figure S2A), nor were uNOS-lir neurites detected in the neuropil of the CPG, but uNOS-lir neurites were detected in the neuropil of the PG (Supplementary Figure S2A1), and uNOS-lir cell bodies were detected in the ventro-central lobes of the VG (Figure 5 Panel A,A1). uNOS-lir nerve fibers were also detected in the neuropils of the VG, the cerebrovisceral connectives, and the fibers extending from the VG (Figure 5 Panel A,A2). After 1 h of hypoxia exposure, the number of uNOS-lir cells increased in the VG compared to that of the control (Figure 5B), and aggregations of neurons in the ventro-central lobe (Figure 5 Panel B1) and solitary neurons in the dorso-central lobe (Figure 5B2) were more clearly labeled.

However, after 3 h of hypoxia, the number of uNOS-lir cells and fibers decreased in the ventro- and dorso-central lobes of the VG (Figure 5 Panel C,C1). By 6 h, no uNOS-lir cells were detected, and immunopositive uNOS-lir fibers were only weakly visible in the VG (Figure 5 Panel D,D1). Regarding NOS, pronounced effects were recorded only at the initial stages of hypoxia (1 and 3 h), whereas after 6 and 12 h, the cells were no longer present.

An estimate shows that the number of uNOS-lir cells sharply increased after 1 h of hypoxia exposure (46.66 ± 2.20 cells) and decreased after 6 h (2.11 ± 0.63 cells) compared to that in the control (11.88 ± 0.84 cells) (Figure 5E). In the CPG, the number of uNOS-lir fibers in the neuropil increased after 3 h of hypoxia exposure, and a slight increase in the staining intensity of uNOS-lir in the neuropil was observed in the PG (Supplementary Figure S2B,B1). After 6 h of hypoxia exposure, the number of uNOS-lir fibers in the CPG neuropil continued to increase, while the number of uNOS-lir fibers in the CPG neuropil varied, but not significantly (Supplementary Figure S2C,C1). The qualitative data on ganglion immunostaining have been confirmed by calculations of CTCF. The CTCF in the lateral regions of the CPG increased compared to that of the control (101.10 ± 9.75) after 1 h (312.28 ± 27.21), 3 h (428.52 ± 46.59), and 6 h (601.88 ± 13.05) of hypoxia exposure (Supplementary Figure S2D). In contrast, no significant changes in fluorescence intensity were detected in the PG after the same length of hypoxia exposure (control, 370.33 ± 31.89; 1 h of hypoxia exposure, 340.39 ± 40.31; 3 h of hypoxia exposure, 437.23 ± 14.24; 6 h of hypoxia exposure, 404.25 ± 38.94) (Supplementary Figure S2E).

Thus, hypoxia in the scallop caused an increase in the number of uNOS-lir cells, which reached their maximum number within 1 h of oxygen removal, while further exposure led to a decrease in the number and, ultimately, the complete absence of uNOS-lir cells in the VG. As for the CPG, there was a steady increase in the number of uNOS-lir fibers between 1 and 6 h. Such a change was not observed in the PG, where the fluorescence intensity value did not vary as dramatically.

#### 2.3.4. FMRFamide-lir Distribution after Hypoxia Exposure

**Figure 6:**
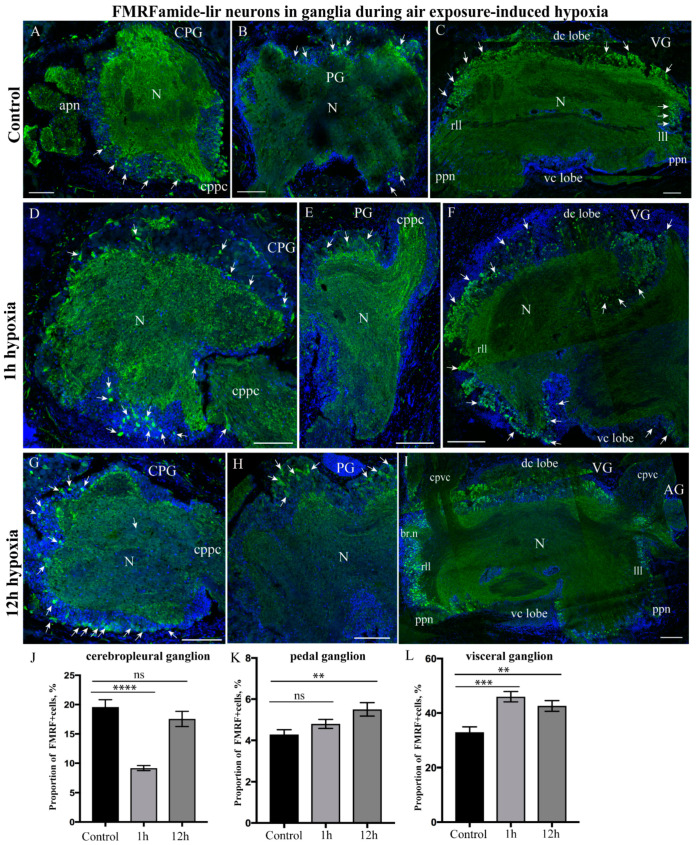
FMRFamide-lir distribution in all control ganglia and during air exposure-induced hypoxia. FMRFamide-lir cells and neurites in the control CPG (**A**), PG (**B**), and VG (**C**). FMRFamide-lir in ganglia after 1 h of hypoxia exposure in the CPG (**D**), PG (**E**), and VG (**F**). FMRFamide-lir in ganglia after 12 h of hypoxia exposure in the CPG (**G**), PG (**H**), and VG (**I**). The arrows on the figures indicate immunopositive cells. Quantitative analysis of immunopositive reaction in the CPG (**J**), PG (**K**), and VG (**L**) under control conditions and after hypoxia exposure (1 and 12 h). The letter designations are as follows: CPG, cerebropleural ganglia; PG, pedal ganglia; VG, visceral ganglia; N, neuropil; vc lobe, ventro-central lobe; dc lobe, dorso-central lobe; lll, left lateral lobe; rll, right lateral lobe; cpvc, cerebral-pleural visceral connectives; ppn, posterior pallial nerves. Data analysis was performed with GraphPad Prism 7; the data are expressed as mean ± SEM, and statistical significance was calculated using one-way ANOVA tests. Multiple comparisons were performed using Dunnett’s many-to-one test (normoxia group was set as the control). Regarding the proportion of FMRFamide-lir cells in the CPG: F = 28.19, *p* < 0.0001 (****), R square = 0.7102, F (DFn, DFd): F (2, 23) = 28.19; in the PG: F = 5.395, *p* = 0.0116 (**), R square = 0.3101, F (DFn, DFd): F (2, 24) = 5.395; and in the VG: F = 12.16, *p* = 0.0002 (***), *p* = 0.0033 (**), R square = 0.5034, F (DFn, DFd): F (2, 24) = 12.16. The degree of significance is represented as follows: **** *p* < 0.0001, *** *p*-value ≤ 0.001, ** *p*-value ≤ 0.01; ns—non-significant *p*-value. Bars: (**A**,**B**,**D**,**E**,**G**,**H**), 50 μm; (**C**,**F**,**I**), 100 μm.

In the control *A. farreri* scallops, FMRFamide-lir neurons and nerve fibers were present in all the cortexes of all three ganglia (Figure 6). The most intensely stained zones were detected in the CPG, especially near the cerebropedal connectives (Figure 6A). In the PG, FMRFamide-lir neurons were identified in all regions of the ganglia; furthermore, fibers were identified in the neuropil, cerebropedal connectives, and pedal nerves (Figure 6B). FMRFamide-lir was detected in all lobes of the VG (Figure 6C). Furthermore, FMRFamide-lir fibers were found in the nerves extending from the ganglion (Figure 6C).

Under hypoxic conditions, it is difficult to estimate the number of FMRFamide-lir cells, as the peptide was expressed in the cortex by a large number of cells in all scallop ganglia after both 1 h and 12 h of hypoxia exposure (Figure 6D–I). As for FMRFamide, 3 and 6 h of hypoxia had no effect on its distribution compared to 1 h of hypoxia. However, a quantitative assessment of the proportion of immunopositive cells (the percentage of ganglion cells which are immunopositive) provides valuable information about the dynamics of the FMRF neuropeptide after both short- and long-term exposure to hypoxic conditions (Figure 6J–L). Following this approach, it was found that after 1 h of hypoxia exposure, the proportion of FMRFamide-lir neurons relative to the number of all neurons in the CPG decreased (9.17 ± 0.41%), and that, after 12 h, the proportion of cells increased (17.54 ± 1.14%) and almost reached the control level (19.58 ± 1.26%) (Figure 6J). In the PG, the proportion of FMRFamide-lir cells increased only slightly (control, 4.28 ± 0.23%; 1 h of hypoxia exposure, 4.79 ± 0.22%; 12 h of hypoxia exposure, 5.50 ± 0.32%) (Figure 6K). Similarly, the percentage of FMRFamide-lir neurons in the VG slightly increased, especially in the dorso-central lobes after 1 h and in the lateral and central regions after 12 h of hypoxia exposure (control, 32.94 ± 1.97%; 1 h of hypoxia exposure, 45.99 ± 1.87%; 12 h of hypoxia exposure, 42.62 ± 1.97%) (Figure 6L).

Thus, in the experimental scallops, hypoxia led to a decrease in the number of FMRFamide-lir neurons in CPG and the restoration of this number to the control level after 12 h of exposure. In general, hypoxia had only a slight effect on the variation in the number of FMRFamide-lir neurons in the VG and increased only slightly in the PG.

### 2.4. Colocalization of Neurotransmitters after Hypoxia

The distributions of 5-HT immunostaining were compared using labeling for CHAT, uNOS, and FMRFamide to detect possible co-localizations in all ganglia in the control and hypoxia exposure groups.

#### 2.4.1. FMRF/5-HT-lir

**Figure 7:**
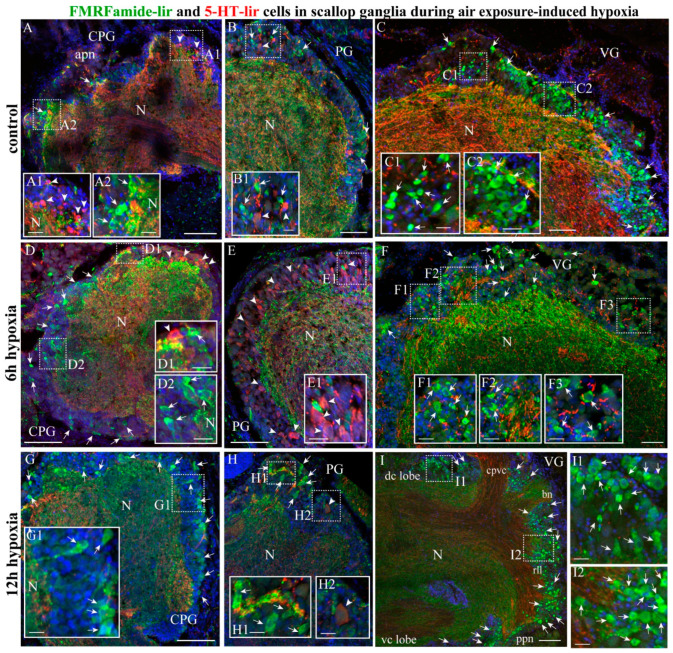
Co-distribution of neurotransmitters FMRFamide and 5-HT-lir during air exposure-induced hypoxia. The distribution of FMRFamide and 5-HT in the CPG (**A**–**A2**), PG (**B**,**B1**), and VG (**C**–**C2**) in the control. The distribution of neurotransmitters after 6 h of hypoxia exposure in the CPG (**D**–**D2**), PG (**E**,**E1**), and VG (**F**–**F3**) and after 12 h of hypoxia exposure in the CPG (**G**,**G1**), PG (**H**–**H2**), and VG (**I**–**I2**). The arrows indicate FMRFamide-lir cells, and the arrowheads indicate 5-HT-lir cells. The letter designations are as follows: AG, accessory ganglia; vc lobe, ventro-central lobe; dc lobe, dorso-central lobe; N, neuropil; lll, left lateral lobe; rll, right lateral lobe; cpvc, cerebral–pleural visceral connectives; bn, branchial nerves; ppn, posterior pallial nerves. Bars: (**A**–**F**), 50 μm; (**A1**–**I2**), 10 μm.

As FMRF and 5-HT were actively expressed in all ganglia or their regions in the scallops, we analyzed the co-localization of these two transmitters in all ganglia (Figure 7). The double immunolabeling of FMRFamide and 5-HT showed that FMRFamide and 5-HT-lir neurons formed a mixed population in the cerebral ganglia of *A. farreri* under normoxic conditions (Figure 7A). However, it was mostly 5-HT-lir cells that were detected in the anterior lobe of the cerebral ganglia and mainly FMRFamide-lir cells in the posterior lobe (Figure 7 Panel A–A2). In the PG, FMRFamide and 5-HT-lir neurons were distributed relatively evenly in all regions (Figure 7 Panel B,B1). The VG exclusively contained FMRFamide-lir neurons in all its lobes, and 5-HT-lir was only detected in nerve fibers in the neuropil, cerebrovisceral connectives, and nerves (Figure 7 Panel C–C2).

After 6 h of hypoxia exposure, the FMRFamide-lir and 5-HT-lir neurons in the cerebral ganglia formed separate populations, the fibers of which constituted the ganglion neuropil (Figure 7 Panel D–D2). This was also observed in the PG (Figure 7 Panel E,E1). Owing to the substantial reduction in the 5-HT-lir fibers in the VG, the FMRFamide-lir neurons of the cortex and the high content of the peptide in the neuropil became most pronounced after 6 h of hypoxia exposure (Figure 7 Panel F–F3). After 12 h, 5-HT-lir levels continued to decrease consistently in the neurons and nerve fibers of the CPG, PG, and VG (Figure 7G–I), whereas FMRFamide-lir immunoreactivity increased in all nerve nodes, with a particularly significant increase in the dorso-central and ventro-central lobes of the VG (Figure 7 Panel I–I2).

Thus, the exposure of scallops to anoxic conditions did not lead to FMRF/5-HT co-transmission in the same neurons; these neuromolecules continued being synthesized in different neurons of the cortex of the ganglia. The general trend previously observed in serotonin levels—a decrease in the amount in the ganglia and the residual detection of this neurotransmitter in the nerves extending from the ganglia—was still observed under these conditions. We did not detect the co-transmission of neurotransmitters in the neurons of any of the three scallop ganglia.

#### 2.4.2. NOS-/5-HT-lir

**Figure 8:**
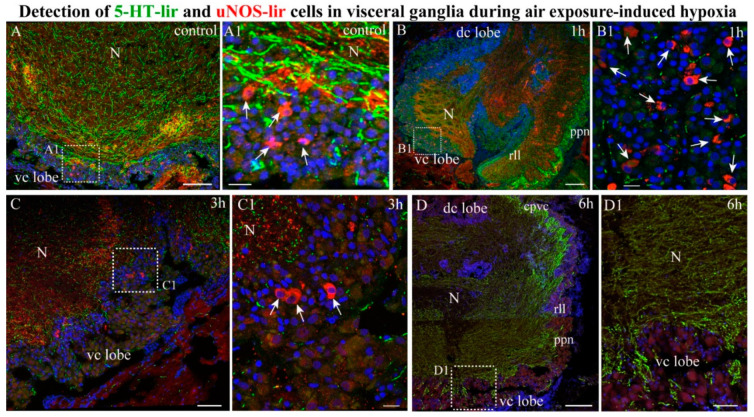
Co-distribution of neurotransmitters NOS and 5-HT-lir during air exposure-induced hypoxia. The distribution of 5-HT and uNOS in control VG (**A**,**A1**), and after 1 h (**B**,**B1**), 3 h (**C**,**C1**), and 6 h (**D**,**D1**) of hypoxia exposure. The arrows indicate uNOS-lir cells. The letter designations are as follows: vc lobe, ventrocentral lobe; dc lobe, dorso-central lobe; N, neuropil; lll, left lateral lobe; rll, right lateral lobe. Bars: (**A**–**D**), 50 μm; (**A1**–**D1**), 10 μm.

As NOS-lir neurons were only detected in the VG, we only performed the double labeling of uNOS and 5-HT in the VG. In the control *A. farreri* scallops, double labeling using immunostaining for uNOS and 5-HT showed that uNOS-lir neurons in the ventro-central lobes were located in close proximity to 5-HT-lir nerve fibers in the VG (Figure 8 Panel A,A1). After 1 h of hypoxia exposure, the population of uNOS-lir neurons, but not of 5-HT-lir fibers, in the ventro-central lobe of the VG increased (Figure 8 Panel B,B1). During this period, uNOS-lir fibers in the VG were mainly localized in the central regions of the neuropil, and 5-HT-lir fibers were detected on the periphery of the ganglion and in the fibers of the pallial nerves (Figure 8B). After 3 h of hypoxia exposure, uNOS-lir was detected in solitary cells, and levels of uNOS- and 5-HT-lir were reduced in nerve fibers (Figure 8 Panel C,C1). After 6 h of hypoxia exposure, no uNOS-lir neurons were detected in the VG, while 5-HT-lir fibers were observed only on the periphery of the ganglion (Figure 8 Panel D,D1). Thus, there was no co-localization of uNOS- and 5-HT in the ganglia. After short-term hypoxia, the number of uNOS cells increased rapidly. After long-term hypoxia, this number rapidly decreased, and serotonin levels in the neuropil fibers decreased and were concentrated to the peripheral fibers of the ganglia.

#### 2.4.3. CHAT/5-HT-lir

**Figure 9:**
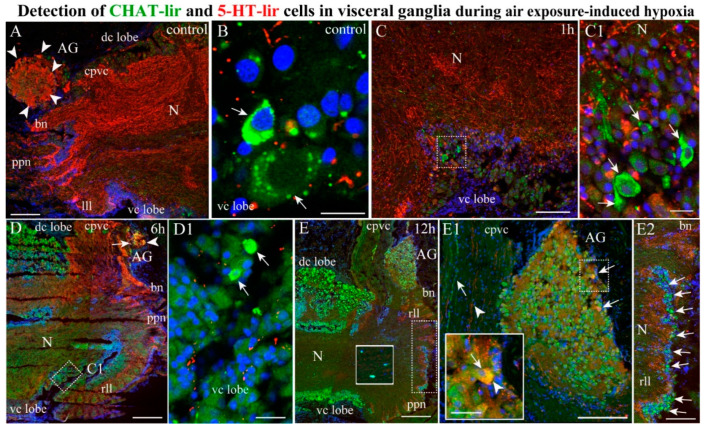
Co-distribution of CHAT and 5-HT-lir during air exposure-induced hypoxia. The distribution of 5-HT and CHAT in control VG (**A**,**B**) and after 1 h (**C**,**C1**), 6 h (**D**,**D1**), and 12 h (**E**–**E2**) of hypoxia exposure. The arrows indicate CHAT-lir cells, and the arrowheads indicate 5-HT-lir cells. The letter designations are as follows: AG, accessory ganglia; vc lobe, ventrocentral lobe; dc lobe, dorso-central lobe; N, neuropil; lll, left lateral lobe; rll, right lateral lobe; cpvc, cerebral–pleural visceral paired connectives; bn, branchial nerves; ppn, posterior pallial nerves. Bars: (**A**–**E**,**E1**), 50 μm; (**B**,**C1**,**D1**), 20 μm.

As the two neuromolecules CHAT and 5-HT showed the most pronounced changes in the scallop ganglia, an analysis of their distribution in the VG was performed. Double labeling using immunostaining for CHAT and 5-HT identified only single CHAT-lir neurons in the VG of the control scallops (Figure 9A,B) and after 1 h of hypoxia (Figure 9 Panel C,C1). These neurons were localized in the ventro-central lobes, near 5-HT-lir fibers (Figure 9 Panel A–C1). After 6 h of hypoxia exposure, the population of CHAT-lir neurons in the dorso-central lobe of the VG increased (Figure 9 Panel D,D1), while 5-HT-lir fibers were detected mainly on the periphery of the ganglion and in the fibers of the pallial and branchial nerves (Figure 9D). At this stage of hypoxia, we detected, for the first time, double CHAT/5-HT-lir in the neurons of the AG (Figure 9D). After 12 h of hypoxia exposure, the CHAT-lir level increased in the dorso-central and ventro-central lobes in the VG, while the 5-HT-lir level decreased significantly in the neuropil fibers (Figure 9E). Furthermore, the CHAT-lir level increased in the neurons of the AG and the lateral lobes of the VG, with a simultaneous decrease in the 5-HT-lir level in the neurons of the AG (Figure 9 Panel E–E2). The double labeling showed that only single neurons had CHAT/5-HT colocalizations in the AG after 12 h of hypoxia exposure. It should be noted that the CHAT and 5-HT-lir fibers in the cerebrovisceral connectives did not colocalize but were oriented in parallel (Figure 9E1). Thus, exposing the scallops to anoxic conditions caused the number of CHAT-lir neurons to increase and the number of 5-HT-lir fibers in the neuropil and entire ganglion to decrease. Moreover, CHAT, which was absent from the AG in the control, began to be expressed in the AG neurons (probably de novo) and was co-localized with 5-HT after 6 h and 12 h of hypoxia exposure.

## 3. Discussion

The anatomical organization of the scallop CNS, considered to be one of the most complex nervous systems among that of bivalves, has previously been studied in detail using light microscopy [58,59,61]. In many respects, scallop neuroanatomy is similar to that of other bivalve mollusks [62]; however, there are some features of their nervous system which are unique.

### 3.1. 5-HT-1ir in Bivalves

The presence of 5-HT in the CNS of *A. farreri* has previously been confirmed by pharmacological, biochemical, and immunocytochemical studies on various bivalve species [19,63,64,65,66]. In the CNS of the control *A. farreri* scallops, the distribution of 5-HT-lir matched the localizations of neurons and nerve fibers in ganglia of other scallop species, such as *Placopecten magellanicus* [64,65] and *Patinopecten yessoensis* [59,61]. In the cerebral ganglia of *A. farreri*, 5-HT-lir is mostly present in the anterior lobes and pallial nerves, which connect to the circumpallial nerves and innervate pallial structures, such as eyes and tentacles [67,68,69].

Similar to *P. yessoensis*, a scallop that has previously been studied, 5-HT-lir neurons are absent in the VG of *A. farreri* [59] but are highly abundant in fibers of the neuropil and branchial nerves, which innervate the gills. The increase in the 5-HT-lir level in the branchial nerves of *A. farreri* after 6 h of hypoxia exposure agrees with the long-established fact that 5-HT has a cilio-excitatory and metabolic stimulatory effect on the gills of several bivalve mollusks [70,71,72]. Increased ciliary and heartbeat rates [73], as well as widening of the inter-lamellar blood vessel by contraction of muscles in the inter-lamellar connections [74], are central mechanisms by which mollusks can stabilize respiratory rates in response to declining oxygen availability [75].

The involvement of serotonin in hypoxia adaptation in invertebrates has been confirmed by previous experimental studies. In encapsulated embryos of the gastropod *Helisoma*, specific serotonergic neurons mediate the hypoxia-induced increase in ciliary beat frequency [76,77]. This induces the rapid rotation of the embryos and more efficient oxygen diffusion due to increased stirring. Similar cilio-excitatory effects have been investigated in experiments on crustaceans, which showed that 5-HT could increase the rate of scaphognathite movement. This ultimately increases water circulation [78], thus promoting more rapid oxygen exchange in tissues as a response to increased oxygen demand. Serotonin is an important mediator of hypoxic responses in oxygen-sensitive pulmonary neuroepithelial bodies and carotid body type I cells in mammals [79,80].

Our data on the decrease in levels of 5-HT in the ganglion neuropil and the increase in its enzyme activity in the mantle and branchial nerves, in the case of hypoxia exposure are consistent with those of studies [81] in which the 5-HT concentration increased significantly in the hemolymph and mantle of Pacific oysters, *Crassostrea gigas*, after exposure to air for 24 h [81]. The substantial decrease in the 5-HT-lir level in the *A. farreri* CNS after 12 h of hypoxia exposure indicates a reduction in the functional activity of the 5-HT system in the scallop ganglia.

In *A. farreri*, as in *Pecten maximus* and *P. yessoensis*, 5-HT-lir neurons were detected in the AG [60,61,82]. This shows that, under the effect of environmental signals (basically, this is an increase in temperature), the osphradial sensory cells (which induce neurosecretory activity in the cells of the osphradial ridge) transport neurosecretions to the AG. This influences the axonal transport of serotonin-like monoamines to the gonad via the gonad nerve [82]. In mollusks, serotonin is known to be involved in the regulation of reproductive function [60,61,83], as it is a potent spawning inducer in bivalves, including pectinids [60,61,84,85,86].

### 3.2. CHAT-lir in Bivalves

**Figure 10:**
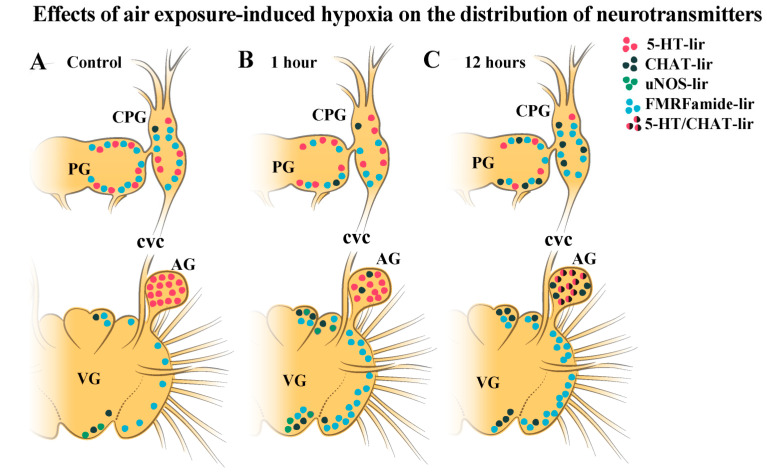
Diagram of dynamic distribution of neurotransmitters in scallop ganglia during air exposure-induced hypoxia. The distribution of neurotransmitters and their quantitative representation are shown for the main scallop ganglia under control conditions (normoxia, (**A**)) and after 1 h (**B**) and 12 h (**C**) of hypoxia exposure in the CPG, PG, and VG. The figure shows that during hypoxia, there was a decrease in the amount of 5-HT in the CPG and PG, and double immunopositive 5-HT/CHAT-lir cells appeared in the VG throughout the experiment (12 h). During short-term hypoxia, there was an increase in the number of uNOS cells in the VG, but no uNOS cells were observed at 12 h after hypoxia exposure. Levels of the peptide FMRFamide increased slightly in the PG and VG and decreased slightly in the CPG. The letter designations are as follows: CPG, cerebropleural ganglia; PG, pedal ganglia; VG, visceral ganglia.

In the VG of the scallop *A. farreri*, CHAT-lir was expressed in large single neurons of the central part of the dorso-central lobe (Figure 10), where the bodies of motor neurons associated with the nerves supplying the adductor muscle are located [67].

Under hypoxic conditions, the CHAT-lir level increased in the central part of the dorso-central lobe of the VG (Figure 10), where the motor neurons involved in the regulation of adductor muscle contractions are localized, as was previously established by Stephens [87]. Furthermore, after 6 h of hypoxia exposure, CHAT expression was detected in the lateral part of the dorso-central lobe of the VG. A cobaltous chloride backfilling of the radial pallial nerves showed that the somata of the mantle edge motor neurons are located on the lateral margins of the dorso-central lobes and controls coordinated movements of the mantle edge [66].

The function of CHAT-lir neurons in the lateral and ventro-central lobes in *A. farreri* remains unknown. The lateral lobes are believed to function as optic lobes [88,89]. It has previously been reported that the lateral lobes in the parieto-visceral ganglia process visual information gathered by the mantle eyes [89,90]. Our data showed that CHAT was expressed in the AG after exposure to anoxic conditions but not after exposure to normoxic conditions. It is known that 5-HT-lir sensory AGs are involved in gonad innervation and probably in gonad maturation and sprawling. Previously, receptors for ACh have been identified in the gonad of the scallop *Pecten maximus*, which indicates that there is cholinergic innervation of the gonad wall. It was also found that receptors to ACh may be involved in the development of the gonads of scallops [31]. CHAT-lir fibers were detected in the branchial nerves of *Mytilus edulis*, where all fibers of the branchial nerve were histochemically positive for acetylcholine esterase (unpublished data). CHAT-lir was also identified in the osphradial nerves, which innervate the osphradia. These are located in the mantle cavity, which is in contact with the aquatic environment, is involved in water quality monitoring, and responds to changes in salinity and osmotic pressure, hypoxia, and various chemicals. Furthermore, FMRFa-lir is common in the osphradial neurons of several gastropod species [91].

This study also identified that neurons of the cerebral and pedal ganglia (which showed no histochemical activity in the control) were involved in response to hypoxia exposure. The mechanisms of CHAT activation and the functional role of CHAT in mollusk ganglia are poorly understood.

We could not find data on the activity of CHAT, the enzyme that synthesizes ACh, in invertebrates under hypoxic or anoxic conditions in the literature. However, previous studies on the role of the cholinergic system in the CNS responses to hypoxia in vertebrates showed that 10 min of complete ischemia (bilateral occlusion of the carotid artery) in mice caused choline (Ch) accumulation [92]. The major source of Ch accumulation during ischemia is the hydrolysis of Ch-containing phospholipids and the hydrolysis of phospholipid-derived choline intermediates, with the contribution of ACh to hydrolysis being small [93]. In neurons, Ch is not only the precursor of ACh, but is also rapidly phosphorylated into phosphorylcholine, a phospholipid intermediate [92]. In cholinergic neurons, the phospholipids containing Ch represent a large source of Ch that can be used for ACh synthesis [94]. The administration of Ch is known to increase the ACh level in the brain [92] and enhance ACh synthesis even under conditions of increased release of ACh [95]. It was also found that Ch protects the brain against ischemic stroke [96]. It cannot be ruled out that an increase in immunoreactivity in scallop ganglia after 12 h of hypoxia exposure is associated with a short-term increase in the Ch level.

### 3.3. uNOS in Bivalves

Our data on the presence and distribution of uNOS-lir in the CNS of *A. farreri* are consistent with the previous results of histochemical and immunohistochemical studies on the NO-ergic system in several bivalve species [46,50,97]. In the CNS of *A. farreri*, the number of NO-positive neurons was significantly lower than those in the previously studied bivalve species *Crenomytilus grayanus* and *Modiolus kurilensis* [97]. Our results showed that the expression of uNOS increased in response to hypoxia within 1 h in the fibers of the ventro-central and dorso-central lobes of the VG (Figure 10) and in pallial, branchial, and osphradial nerves. This indicates that NO is involved in the adaptation to hypoxia in mollusks. It has recently been reported that, under hypoxic conditions, NO is generated in the muscle cells surrounding the hemolymph vessels of gill filaments in *M. edulis*, causing an opening of the blood vessels, which functionally stabilizes the respiration of the whole animal as pO_2_ [75]. The increase in NO production can be associated either with the development of compensatory mechanisms aimed at maintaining aerobic processes or with switching to anaerobic metabolic pathways. In most invertebrates, including mollusks, the survival strategy under hypoxic conditions is to reduce overall ATP consumption [98,99]. This ability of mollusks to respond to low oxygen concentrations in water by either maintaining internal energy resources by switching to anaerobic metabolic pathways or by reducing the rate of overall metabolism through metabolic depression has been reported in many works [98,100,101,102]. According to recent studies, NO plays a key role in metabolic rate depression in both vertebrate and invertebrate species when entering a dormant or quiescent state, such as hibernation or torpor, or during exposure to hypoxic or anoxic conditions. Such conditions are usually experienced by animals living in intertidal and subtidal habitats [75]. NO is known to be involved in the regulation of mitochondrial respiration in the cells of vertebrates and invertebrates [75,103]. It is likely that hypoxia triggers the production of NO, which, in NO-ergic neurons of the CNS in mytilids (animals resistant to hypoxia), is involved in a range of processes related to respiration. Such processes include the inhibition of the mitochondrial respiratory pathway and switching neurons from aerobic respiration to glycolysis under conditions of reduced intracellular oxygen concentrations, a process that minimizes the production of reactive oxygen species in the cell [104,105]. This hypothesis has been confirmed by experimental studies on the molecular mechanisms of hypoxia resistance in mytilids [105,106] and *Arctica islandica* [104].

In *A. farreri*, unlike mytilids, the uNOS-lir level in the CNS increased only slightly with hypoxia. This contrasts with the NO-ergic activity in the ganglia [50], muscle cells, and hemolymph vessels of gill filaments in mytilids under both control and hypoxia exposure conditions [75]. The significant differences in NO expression between these bivalve species may be related to their differing lifestyles and strategies of ecological adaptation [104]. In the intertidal zone, mussels are exposed to hypoxia and anoxia during low tide [73,106], which requires fast metabolic adjustment upon shell closure [104]. In *M. edulis*, pO_2_-dependent NO generation is a key mechanism for withstanding rapid environmental oxygen fluctuations [75]. In contrast to slow-moving mollusk species, active scallops are less adapted to hypoxia owing to the high energy cost of movement [107]. Nevertheless, they are capable of escape behavior. These data are consistent with those of studies on the roles of gills, respiratory metabolism, and oxidant enzyme activity in bivalves [108]. For example, *Mytilus* spp. have a response to hypoxia and anoxia characterized by regular reductions in metabolism and the transition to anaerobic energy generation pathways [109,110]. *Pecten maximus*, a different species of mollusk, responds differently. After being exposed to hypoxia or combined conditions of anoxia and elevated temperature for 1–4 h, *P. maximus* increases its respiratory and heart rates to maintain its aerobic metabolism, leading to an acceleration of hemolymph circulation [108]. Compared to *P. maximus*, the scallop *A. farreri* exhibited a low capacity for respiratory regulation [111]. Even in the case of severe hypoxia exposure, the heart rate in Zhikong scallops was only slightly reduced, suggesting a lack of cardiac regulatory ability. An increase in heart activity itself is known to require more energy and, thus, more oxygen. However, according to the data presented by Li [111], the activity of lactate dehydrogenase is significantly activated as the level of dissolved oxygen decreases, which may result in the excessive accumulation of lactate and, possibly, the death of Zhikong scallops under hypoxia. Thus, the increase in the uNOS-lir level in certain neurons of the VG, as well as the branchial, osphradial, and mantle nerves in *A. farreri*, as induced by hypoxia, may reflect its key role in the regulation of gas exchange, osmoregulation, and the activation of the compensatory response in reaction to short-term hypoxia exposure.

### 3.4. FMRFamide in Bivalves

Our findings regarding the distribution of FMRFamide-lir in the CNS of the control *A. farreri* scallops are consistent with the data on the sea scallops *Placopecten magellanicus* and *Pecten maximus* in the literature [112,113]. The tetrapeptide FMRFamide was first identified in the bivalve *Marocallista nimbosa* [114], and the presence of immunologically related substances was subsequently reported in many species, from coelenterates to vertebrates [53]. In *Pecten maximus*, as in many other mollusks, FMRFamide-lir-related substances are abundant. In the scallop *A. farreri*, as well as in *P. magellanicus* [112], a high concentration of FMRFamide-lir was recorded in the lateral lobes of the parieto-visceral ganglia (Figure 10), which, as reported previously, process the visual information gathered by the mantle eyes [89,90,115]. It was demonstrated that FMRFamide was present in the optic lobes of the squid *Loligo pealei* and in *Octopus vulgaris* [116]. In *O. vulgaris*, it was detected in several (optic, subpedunculate, and olfactory) lobes of the CNS, where it integrates and relays the olfactory, chemical, and visual inputs to the optic gland in order to modulate the secretory activity of the latter. The stimulation of the isolated retina from the eyes of *O. vulgaris* eyes revealed that this FMRFamide, coupled with dopamine, induces extreme light adaptation in the retina under illumination [117,118]. In the optic nerve of the marine gastropod *Bulla goldiana*, some efferent fibers also contain FMRFamide-like neuropeptides [119].

The presence of FMRFamide-lir in motor neurons of the dorso-central lobes is consistent with the results of studies identifying the presence of FMRFamide-lir in the neuromuscular junctions [120] and adductor muscles of mollusks. The definitive role of this family of peptides in scallop reproduction should be elucidated in future studies, even if previous results have suggested that their main actions relate to muscular contraction [121]. Furthermore, FMRFamide is known to play a cardioregulatory role in bivalves [53,121,122,123]. Additionally, the regulation of hypoosmotic cell volume by cardiac muscles in the clam *Mercenaria mercenaria* has been found to be potentiated by FMRFamide [124].

In *A. farreri*, FMRFamide-lir was identified in the branchial nerves that innervate the gill. Previously, the presence of FMRFamide-lir in the branchial nerves and gills has been reported for several species of mollusks, [53] both in adult and larval forms. In these organs, FMRFamide-lir can have both a stimulating and inhibitory effect on ciliary beat frequency [77,125,126], which indicates the involvement of FMRFamide peptides in respiration regulation.

High FMRFamide-lir activity has also been recorded in the osphradial nerves. In gastropods, FMRFamide-lir is assumed to perform a chemosensory function [127,128]. Furthermore, there is some evidence that the neuropeptide family associated with FMRFamide performs an antidiuretic role and is involved in osmotic and volume regulation [129,130]. In experiments on the pulmonate snail *Melampus bidentutus*, FMRFamide levels in the CNS, hemolymph, and kidneys were shown to vary under different osmotic conditions, being lower under hypoosmotic than under hyperosmotic conditions [130]. It was found that the FMRFamide levels increased in the CNS, hemolymph, and kidneys in cases of increased water loss.

## 4. Materials and Methods

### 4.1. Experimental Animals and Establishment of Hypoxic Conditions

Adult specimens of scallop *Azumapecten farreri* (58 ± 2 mm in shell length) were captured from Peter the Great Bay (Sea of Japan) at a depth of 2–3 m. The scallops were placed in tanks with filtered seawater under strictly controlled conditions: temperature, 18 ± 0.5 °C; salinity, 31–33%; oxygen concentration in water, 8.1–8.5 mg/L. The animals were fed an algal diet during the 2-week acclimation period prior to experiments. After acclimation, scallops in one group (exposure) with forcibly closed-shell valves were exposed to air in closed humid chambers at a temperature equal to that of seawater [131]. The other group (control) was kept under the same normoxic conditions described above. Hypoxic stress was elicited by exposing the scallops to air at 18 °C for 12 h according to the method of Giannetto [132]. During the air exposure, the scallops were sampled at 0, 1, 3, 6, and 12 h to evaluate the expression of hypoxic marker HIF1alpha, and their nervous ganglia were dissected immediately. The nervous ganglia from the control group were sampled and dissected at the same time. All applicable international, national, and/or institutional guidelines for the care and use of animals were carefully adhered to.

### 4.2. SDS and Western Blotting of HIF1alpha

To determine the time frame of hypoxia, we analyzed the production and dynamics of the main marker of hypoxia, HIF1alpha [132,133]. SDS-PAGE and Western blotting methods were used. Mouse brain extract (2 mg/mL in SDS-PAGE loading buffer, Sigma-Aldrich, St. Louis, MO, USA, B6928) was used as a positive control. The concentration of total protein in the extract of scallop ganglia was determined using the Lowry method [134]. The electrophoresis of proteins was carried out in a 12% polyacrylamide gel. The amount of total protein used for electrophoresis was 30 μg per well. After SDS-PAGE, the proteins were transferred to a nitrocellulose membrane (Sigma-Aldrich, St. Louis, MO, USA). The nonspecific binding of antibodies was blocked by 5% non-fat dry milk, with the addition of 0.1% Tween-20 and incubation overnight at 4 °C. The same solution was used for subsequent dilution of the antibodies. The membrane was incubated with primary mouse monoclonal antibodies against HIF1alpha (ab16066, Abcam, Boston, MA, USA, dilution 1:1000) for 2 h at 4 °C. Glyceraldehyde-3-phosphate dehydrogenase, 37 kDa, (GAPDH, 1:1000, ABS16, Merck, Millipore, United Kingdom) was used as a loading control to verify the even loading of the samples. After washing in Tris-HCl buffer, the membranes were incubated for 2 h in a solution of secondary antibodies (Vector Labs, Burlingame, CA, USA). A VIP Substrate Kit (Vector Labs, Burlingame, CA, USA) was used to visualize the peroxidase reaction.

### 4.3. Antibodies

Rabbit or goat polyclonal anti-serotonin antibodies coupled to bovine serum albumin (BSA) with paraformaldehyde (PFA) (Hudson, WI, USA, ImmunoStar Incorporated, Cat. No. 20080 and 20079) were used. The manufacturer states that staining with these antisera is eliminated completely by pretreatment with 25 μg of the same serotonin–BSA conjugate per 1 mL of diluted antibody. For controls, we showed that the preincubation of the antibody with the same conjugate (10 μg/mL, ImmunoStar, Cat. No. 20081) at 4 °C overnight completely eliminated all immunolabeling of serotonin in the tissues. The preadsorption of the diluted antiserum with 10 mg/mL BSA overnight at 4 °C did not influence this staining, indicating that these antibodies recognized only serotonin and not BSA. Previous data have shown that these antibodies detect serotonin in mollusks [135,136,137,138]. FMRFamide-like peptides were identified in the central and peripheral nervous systems of various taxonomic groups of mollusks [53,54,55,120,123,139]. The antiserum employed here was generated in rabbit against the synthetic FMRFamide Phe-Met-Arg-Phe-amide conjugated to bovine thyroglobulin (Immunostar Incorporated, Cat. No. 20091). According to the published data, this antibody reacts with FMRFamide in various animals, including mollusks (gastropods, bivalves, and poyplacophorans) [136,137,138,139], indicating that the antigen has been conserved evolutionarily across a broad range of species. The manufacturer confirmed, specifically, that these antibodies react with antigens in caenogastropods, chitons, gastropods (*Helix pomatia*, *Aplysia* sp., *Ilyanassa obsoleta*, *Lymnaea stagnalis*, *Mopalia muscosa*, abalones), and bivalves (*Mytilus trossulus*).

Cytoplasmic CHAT, which synthesizes AChE, is a much more specific marker of cholinergic neurons than AChE. In the present study, we used the concentration of antibody to CHAT recommended by the manufacturer, along with a concentrated blocking buffer to eliminate non-specific binding. In addition, to control for non-specific immunorecognition, immunohistochemical staining was carried out without the primary antibodies, in the presence of only the secondary antibodies or normal (non-immunized) immunoglobulin G (1:500–1:1000, Sigma-Aldrich; I5006, I5381, and I5256).

The NOSs identified in gastropods, cephalopods, and bivalves, including scallops [38,39,40,41,42,139], belong to a single isoform. This antibody has been successfully used in Western blotting and immunohistochemistry previously and specifically for procedures performed on invertebrate samples and mollusks [140,141].

### 4.4. Immunohistochemistry

The dissected *A. farreri* ganglia were fixed in a 4% PFA solution in phosphate-buffered saline (PBS) (pH 7.4) at 4 °C for 2–3 h. Fixed samples were washed in PBS five times at 4 °C for 1 h and cryoprotected by incubation at 4 °C overnight in PBS containing 30% sucrose and then embedded in optimal cutting temperature medium and frozen at –20 °C. Neuronal tissues from three to six control and experimental animals were cut into nine representative 20 μm sections, which were mounted on glass slides and stored at −20 °C before immunostaining.

Immunohistochemical analysis was performed on freshly frozen sections, as described previously [136,138]. To eliminate non-specific binding, the samples were incubated in the blocking buffer (5–10% normal donkey serum (Baltimore Pike, PA, USA Jackson ImmunoResearch), 1–2% Triton-X 100 (Sigma), and 1% bovine serum albumin (Sigma) in 1 × PBS) overnight at 4 °C, with the primary antibodies diluted in this blocking buffer.

For immunostaining, the antibodies were used alone or in combination with other antibodies. The primary antibodies used for neurotransmitter detection in control and experimental scallop tissues were anti-goat 5-HT, anti-rabbit 5-HT, anti-rabbit FMRFamide, anti-rabbit uNOS, and anti-goat CHAT alone. For the identification of neurotransmitter colocalization, we used the following antibody mixtures: anti-goat 5-HT with anti-rabbit FMRFamide, anti-rabbit 5-HT with anti-goat CHAT, and anti-goat 5-HT with anti-rabbit uNOS. The secondary antibody mixtures consisted of donkey anti-goat (DAG) 555 or 488 with donkey anti-rabbit (DAR) 488 or 555 (all from Thermo Fisher Scientific, Waltham, MA, USA). All primary antibodies were first incubated with tissue sections overnight at 4 °C, followed by three rinses with PBS containing 0.1% Tween-20. Then, the sections were incubated with the secondary antibodies for 2 h at RT. The mixtures of secondary antibodies also contained 4′,6-diamidino-2-phenylindole (DAPI, Sigma-Aldrich, St. Louis, MO, USA). The slices were then mounted on glass slides with glycerol-based media (Merck & Co., Kenilworth, NJ, USA). As a control for non-specific immunorecognition, we performed immunohistochemical staining without primary antibodies and added only the secondary antibodies or normal (non-immunized) immunoglobulin G (1:500–1:1000; Sigma-Aldrich, St. Louis, MO, USA; I5006, I5381, and I5256). To determine the localization and dynamics of 5-HT-, CHAT-, uNOS-, and FMRF-lir in the nerve ganglia, experiments were set up with a single label (Figure 3, Figure 4, Figure 5 and Figure 6). Double-labeling experiments were performed to observe the colocalizations of 5-HT-lir with uNOS-lir, CHAT-lir, or FMRFamide (Figure 7, Figure 8 and Figure 9).

### 4.5. Microscopy and Imaging

All images were taken using a Zeiss LSM 780 confocal microscope (Far Eastern Center of Electron Microscopy, A.V. Zhirmunsky National Scientific Center of Marine Biology, Far Eastern Branch of the Russian Academy of Sciences, Vladivostok, Russia) and processed and analyzed using the Imaris (Bitplane, Zurich, Switzerland) and ImageJ (National Institutes of Health, Bethesda, MD, USA) software. The latter software was also used for the three-dimensional visualization and analysis of images. The presented figures show the projections of maximum immunoreactivity.

### 4.6. Quantification and Statistical Analysis

Consecutive series of sections from ganglia tissues were examined. To quantify fluorescence, nine images were taken for each control and hypoxic ganglion with the same scan settings. We here use the terms “staining intensity” and “level” to describe the relative amounts of immunoreactive staining in positive cells or fibers when visually compared to the control. When these terms are used to describe the immunoreactive staining of different antigens, they refer to the fluorescence intensity resulting from the immunochemical procedures. Scallop ganglia sections used for immunohistochemistry and quantitative evaluation were strictly from the same regions. This allowed us to accurately compare the distribution of immunoreactive neuromolecules in tissues. For fluorescence intensity quantification, the immunostained neuronal tissues were processed using ImageJ 1.51w image processing software [142,143]. Each photograph was imported into ImageJ, and the images were adjusted to the threshold using the method of Otsu (1979) [144], and the area and mean fluorescence of the foreground (signal of Alexa 488) and the background across the entire image were measured. The corrected fluorescence was calculated according to the formula created by Luke Hammond (QBI, The University of Queensland, Australia, https://theolb.readthedocs.io/en/latest/imaging/measuring-cell-fluorescence-using-imagej.html) from 10 August 2021. 

CTCF signal = mean fluorescence of the foreground − (foreground area × mean fluorescence of the background).

The areas and numbers of cells expressing specific molecular markers were measured in nine representative sections from each of the two whole CPG, the one fused PG, and the VG of at least three scallops (biological n = 3–6, technical n = 9). Only cells with visible nuclei were counted. All quantifications were performed using ImageJ software, and the values were processed using Prism 7 software (GraphPad, San Diego, CA, USA). The results are presented as mean values ± standard error of the mean for each ganglion and type of staining. Values of *p* < 0.05 were considered to be statistically significant.

## 5. Conclusions

In the present study, we discovered that the exposure of *A. farreri* to hypoxia causes changes in the expression of neurotransmitters (FMRFamide peptide and monoamine 5-HT) and enzymes (CHAT and uNOS) involved in ACh and NO synthesis in the ganglia of the CNS. Our results show that the localization and levels of synthesis of the tested neuromolecules change, and the number of cells expressing these molecules in the scallop CNS varies, during hypoxia, which indicates their possible involvement in hypoxia-resistance mechanisms. The results found here indicate that the reduction in 5-HT and uNOS levels and the increase in the number of CHAT-lir cells after long-term stress exposure may be associated with the release, degradation, and/or accumulation of these enzymes. This suggests that these neurotransmitters perform a range of different physiological functions in mollusks under stressful conditions. Thus, the increase in the number of CHAT-positive neurons in the ganglia may be explained by the short-term increase in the Ch level there, indicating the cholinergic system of the CNS plays an important role in responding to hypoxia. Of particular interest are the variations in the levels of 5-HT, CHAT, uNOS, and FMRF in certain neurons of the VG, as well as in the branchial, osphradial, and mantle nerves. This indicates that they may play a key role in the regulation of gas exchange, osmoregulation, and the activation of compensatory response to short-term hypoxia under conditions of restricted mobility in mollusks.

## Data Availability

The datasets used and analyzed during the current study are available from the corresponding author on reasonable request.

## Supplementary Materials

The following are available online at https://www.mdpi.com/article/10.3390/ijms23042027/s1.
