# Supplementary material for: Effect of Air Exposure-Induced Hypoxia on Neurotransmitters and Neurotransmission Enzymes in Ganglia of the Scallop Azumapecten farreri
Source: Int J Mol Sci. 2022 Feb 11;23(4):2027. doi: 10.3390/ijms23042027 (PMC8878441; doi:10.3390/ijms23042027)
Supplement: Supplementary file 1 [file ijms-23-02027-s001.zip › ijms-1522529 - Supplementary Material/Supplementary Materials.pdf]

Supplementary Materials

CHAT-lir neurons in CPG and PG ganglia during air exposure-induced hypoxia

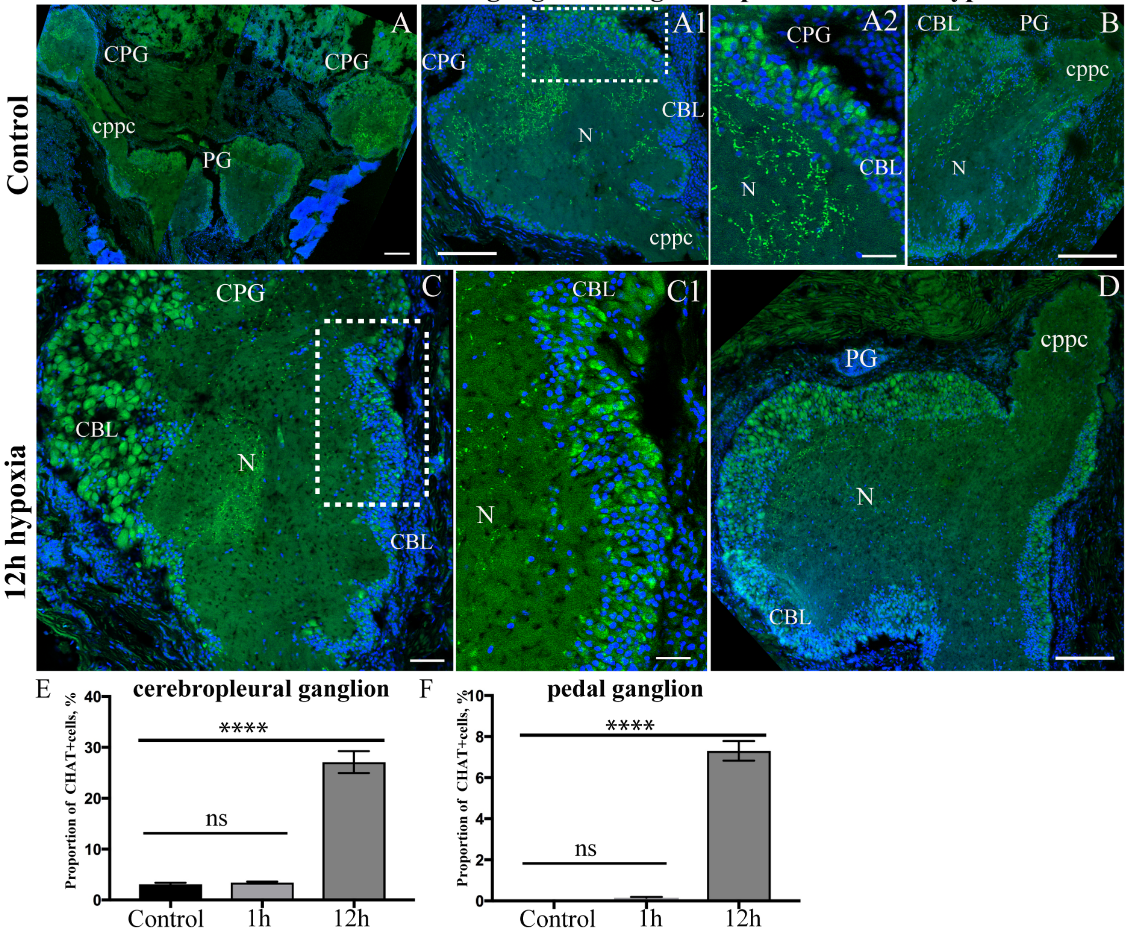

Figure S1. Choline acetyltransferase-lir distribution in control cerebropleural and pedal ganglia and after hypoxia exposure. The distribution of CHAT-lir in the CPG (A, A1, A2) and PG (B) under control conditions and after 12 h of hypoxia exposure (C, C1, D). Quantification of CHAT-lir cells in the CPG (E) and PG (F) under control conditions and after hypoxia (1 h and 12 h). Data analysis was performed using GraphPad Prism 7; the data are expressed as mean ± SEM, and statistical significance was calculated using one-way ANOVA tests. Multiple comparisons were performed using the Dunnett's many-to-one test (normoxia group was set as the control). Regarding the proportion of CHAT+ cells in CPG: $F = 119$, $P = 0.9774$ (ns), and $P < 0.0001$ (****), R square = 0.9084, F (DFn, DFd): F (2, 24) = 119. Regarding the proportion of CHAT+cells in PG: $F = 225.1$, $P = 0.8991$ (ns), and $P < 0.0001$ (****), R square = 0.9494, F (DFn, DFd): F (2, 24) = 225.1. The letter designations are as follows: CPG, cerebropleural ganglia; PG, pedal ganglia; cppc, cerebral–pleural–pedal connectives; N, neuropil; CBL, cell body layer. Bars: A, 500 μm; A1, B, and D, 100 μm; A2, C, and C1, 50 μm.

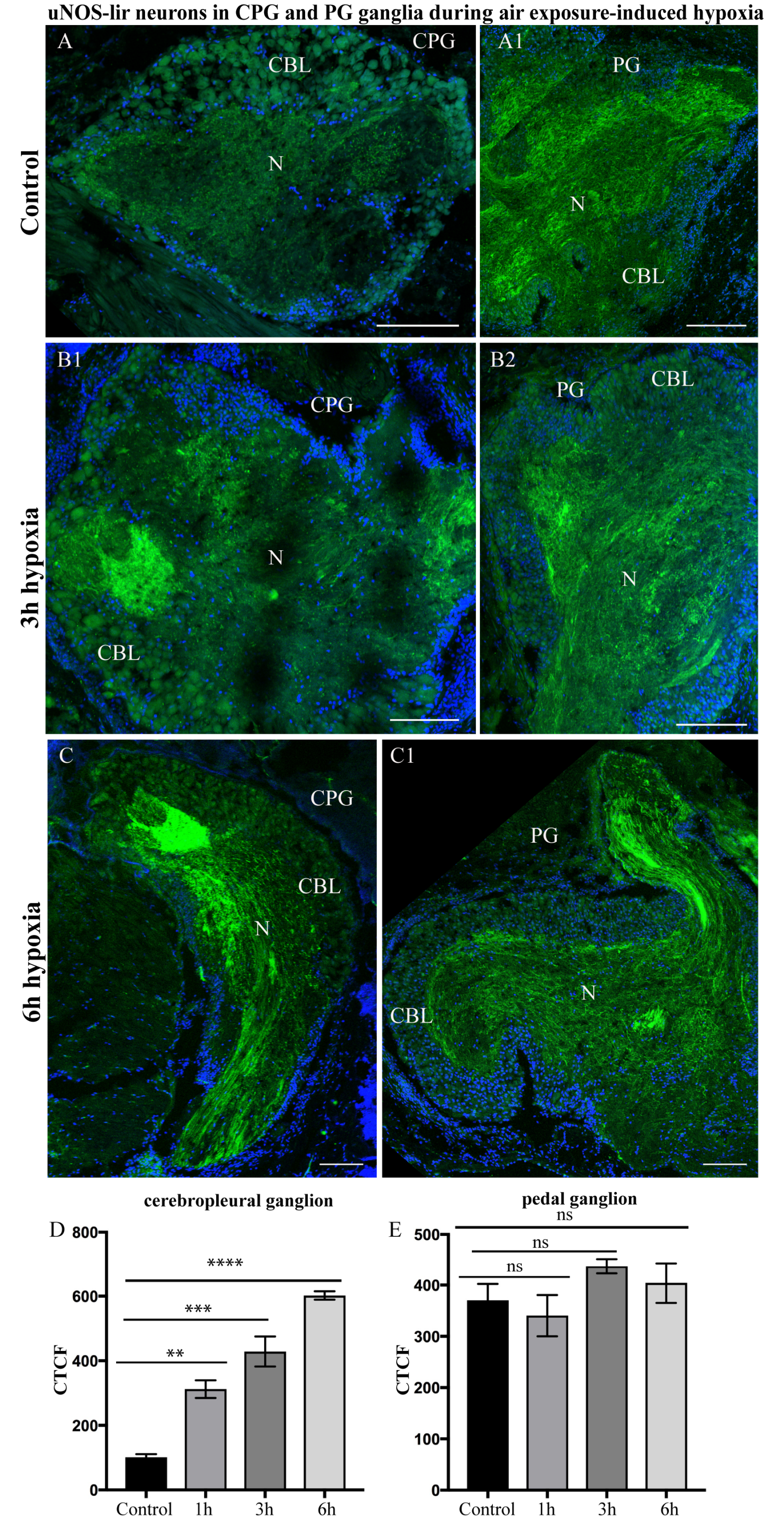

Figure S2. uNOS-lir distribution in control cerebropleural and pedal ganglia and after hypoxia exposure. The distribution of uNOS-lir neurons in control CPG (A), PG (A1), and after 1 h (B1, B2) and 6 h of hypoxia exposure (C, C1). (E) Quantification of uNOS-lir in the CPG (D) and PG (E). The letter designations are as follows: CPG, cerebropleural ganglia; PG, pedal ganglia; N, neuropil; CBL, cell body layer. Data analysis was performed using GraphPad Prism 7; the data are expressed as mean ± SEM, and statistical significance was calculated using one-way ANOVA tests. Multiple comparisons were performed using the Dunnett's many-to-one test (normoxia group was set as the control). With regard to the CTFC in the CPG ganglia after hypoxia, $F = 55.61$, $P = 0.0019$ (**), $P = 0.004$ (***), $P = 0.0001$, R square = 0.7958, F

(DFn, DFd): $F (2, 24) = 46.78$. For the PG after hypoxia, $P = 0.2622$ (ns), R square = 0.3764, F (DFn, DFd): $F (3, 8) = 1.61$. Bars: A, B1, C, and C1, 100 µm; A1 and B2, 50 µm.
