# Supplementary figures and images for: Effect of Air Exposure-Induced Hypoxia on Neurotransmitters and Neurotransmission Enzymes in Ganglia of the Scallop Azumapecten farreri
Source: Int J Mol Sci. 2022 Feb 11;23(4):2027. doi: 10.3390/ijms23042027 (PMC8878441; doi:10.3390/ijms23042027)

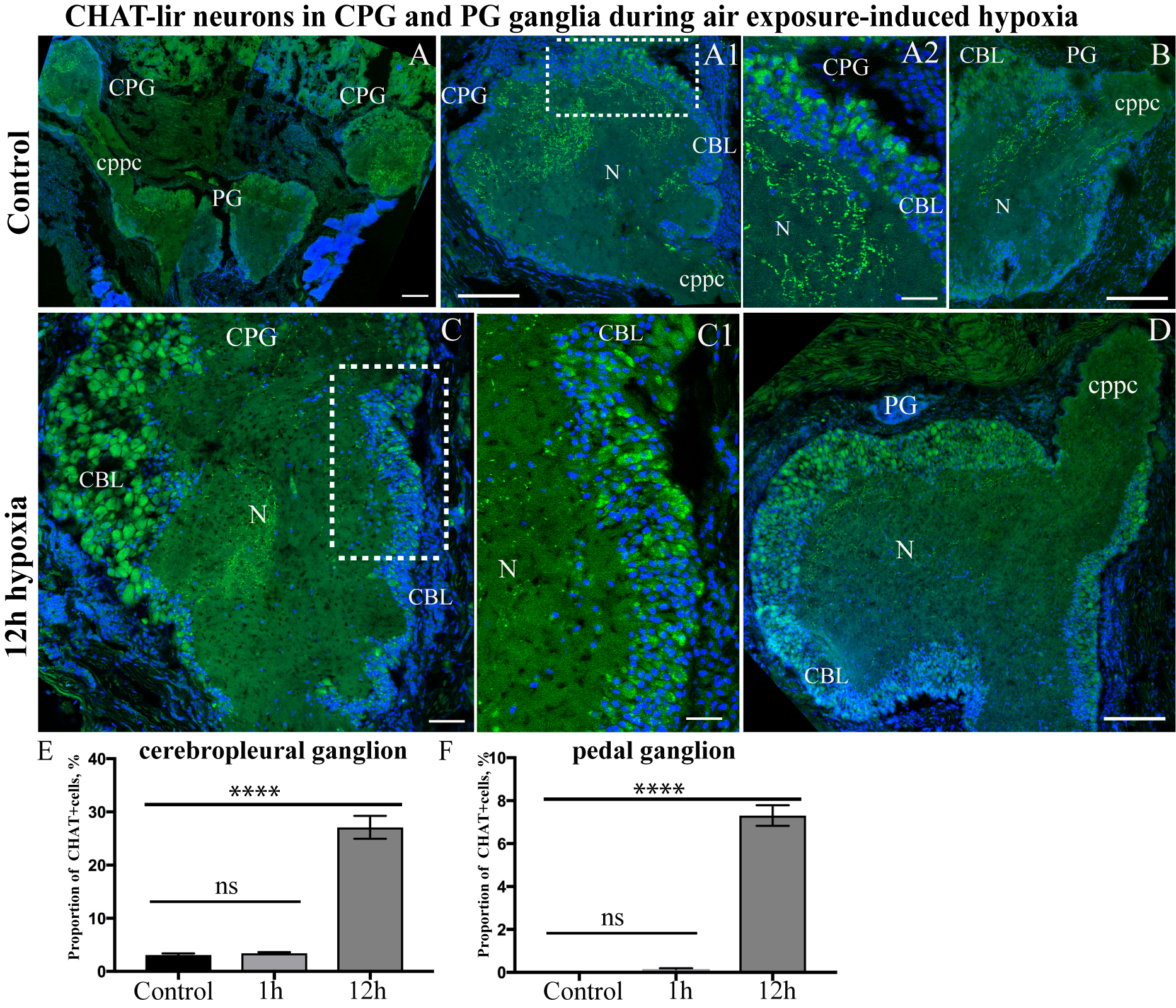

Supplement: Supplementary file 1 [file ijms-23-02027-s001.zip › ijms-1522529 - Supplementary Material/Fig.Supp1.tif]

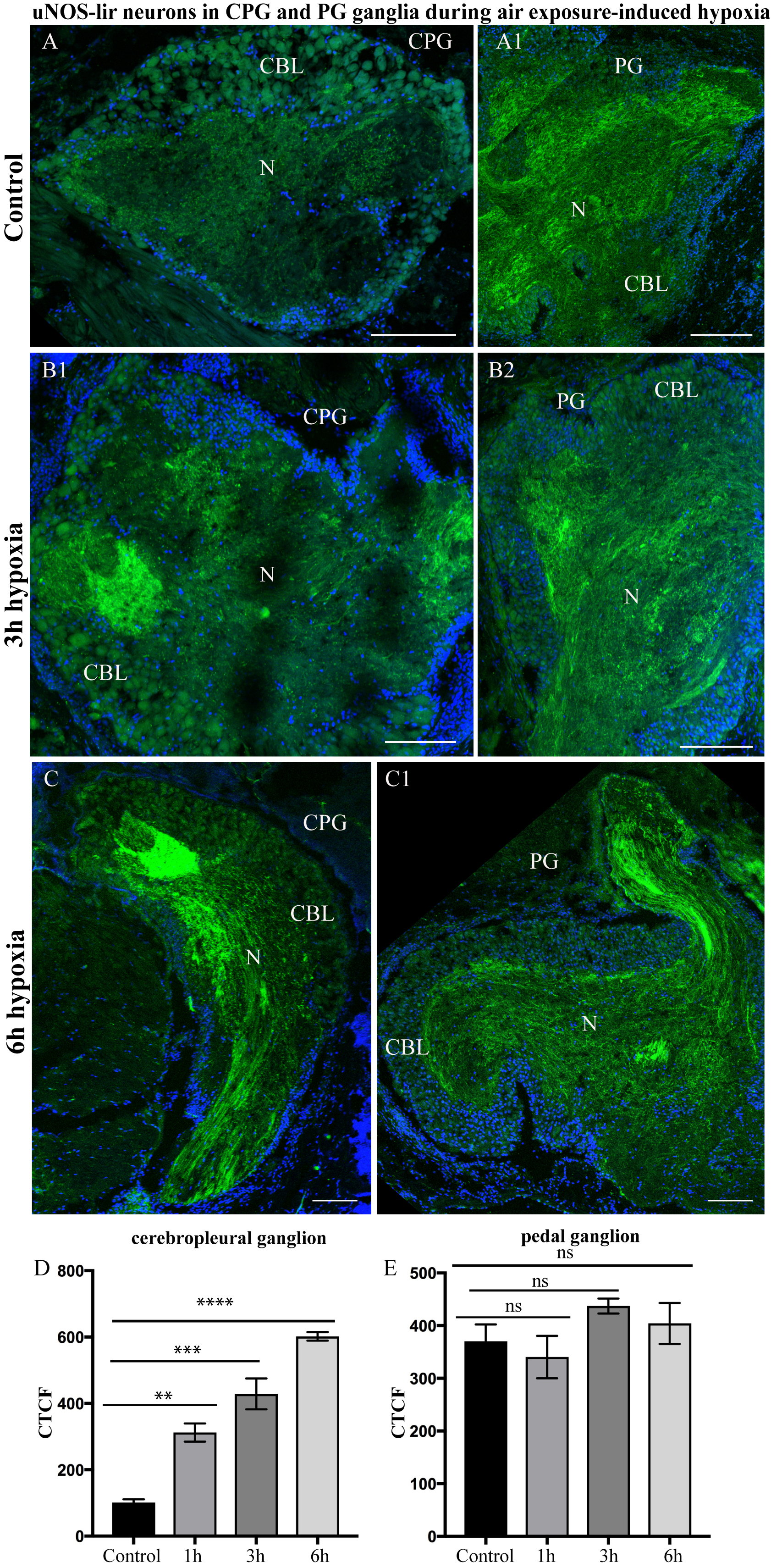

Supplement: Supplementary file 1 [file ijms-23-02027-s001.zip › ijms-1522529 - Supplementary Material/Fig.Supp2.tif]
